# Supplementary material for: Classification at the accuracy limit: facing the problem of data ambiguity
Source: Sci Rep. 2022 Dec 21;12:22121. doi: 10.1038/s41598-022-26498-z (PMC9772417; doi:10.1038/s41598-022-26498-z)
Supplement: Supplementary file 1 — Supplementary Information. [file 41598_2022_26498_MOESM1_ESM.pdf]

SUPPLEMENTAL MATERIALS

for

Classification at the Accuracy Limit - Facing the Problem of Data Ambiguity

Claus Metzner[1,2], Achim Schilling[1,3], Maximilian Traxdorf[4], Konstantin Tziridis[1], Andreas Maier[5], Holger Schulze[1], and Patrick Krauss[1,3,5,*]

[1]Neuroscience Lab, University Hospital Erlangen, Germany
[2]Biophysics Lab, Friedrich-Alexander University Erlangen-Nuremberg, Germany
[3]Cognitive Computational Neuroscience Group, Friedrich-Alexander University Erlangen-Nuremberg, Germany
[4]Department of Otorhinolaryngology, Head and Neck Surgery, Paracelsus Medical University, Nuremberg, Germany
[5]Pattern Recognition Lab, Friedrich-Alexander University Erlangen-Nuremberg, Germany
[*]Corresponding author

November 9, 2022

References

[1] J. Salamon, C. Jacoby, and J. P. Bello. A dataset and taxonomy for urban sound research. In *22nd ACM International Conference on Multimedia (ACM-MM'14)*, pages 1041–1044, Orlando, FL, USA, Nov. 2014.

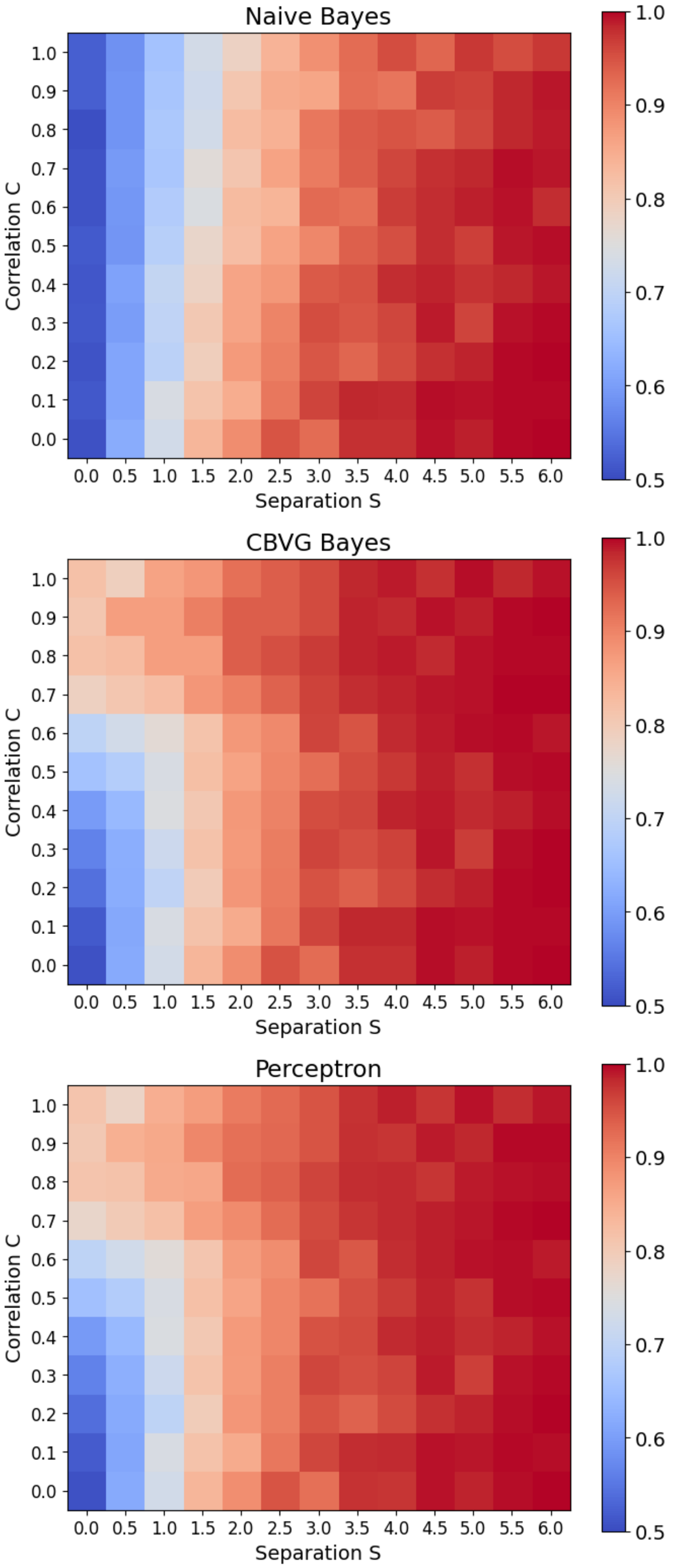

Figure 1: Accuracy of the three classifier models in the DSC data model, when the separation S and the correlation C are simultaneously varied, at a constant dimensionality of D=5. The colors in the heatmap for each (S,C) parameter combination represent the average over 10 independent runs. The CBVG Bayes model and the Perceptron yield almost identical results, indicating that they both have reached the theoretical accuracy limit for this classification problem.

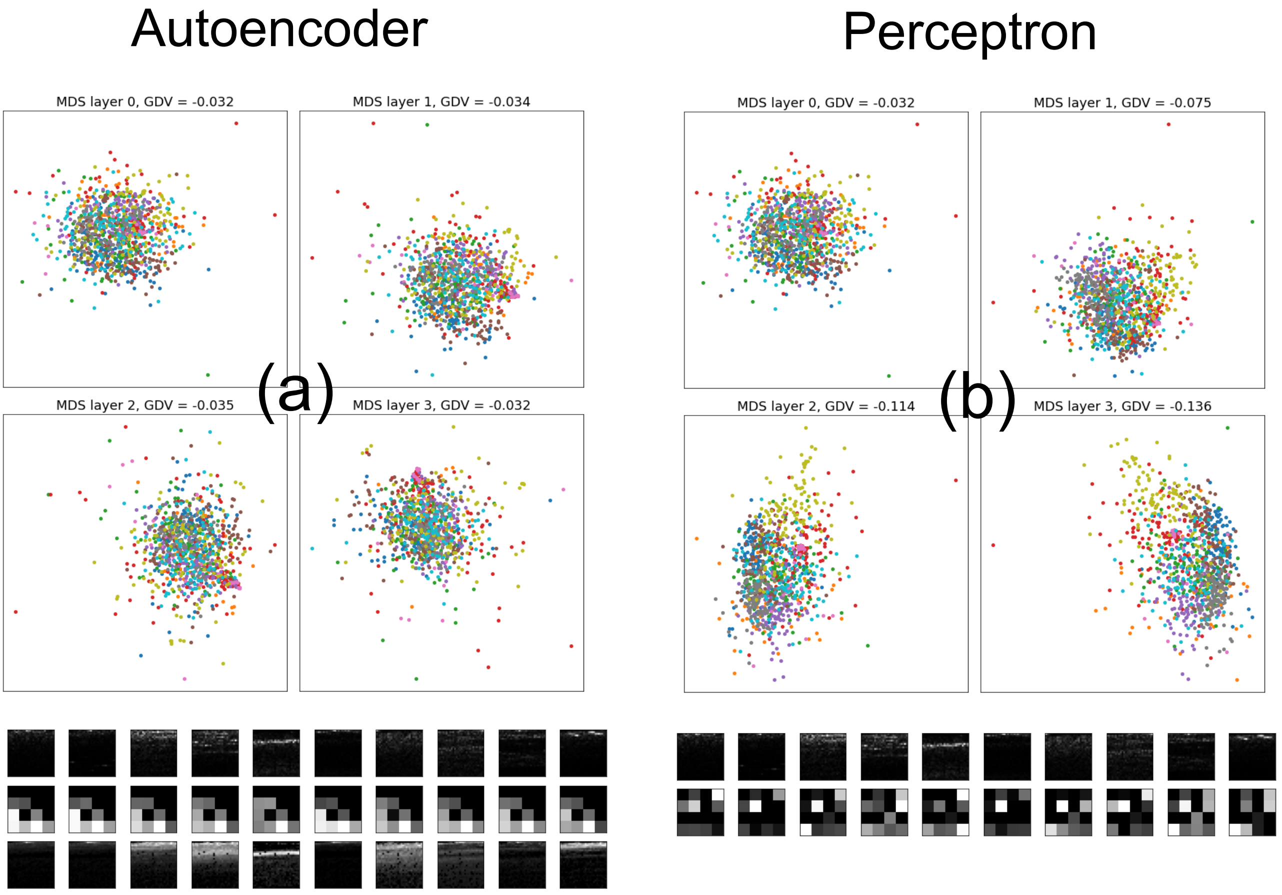

Figure 2: Clustering of natural data distributions in different network layers, after supervised and unsupervised training. While we have analyzed MNIST and sleep EEG data in Figure 9 of the main paper, we here analyze the Fourier amplitudes of sound data. They are recorded in cities and fall into 10 human-defined categories, such as sounds from reconstruction, cars, or children [1]. The original 'Urban Sounds' data set consists of 8732 individual files of different lengths (typically 4 seconds) and formats. We first convert them to mono, a uniform sample rate of 44100 per second, and a standard PCM coding. Discarding all files with a length of less than 0.2 seconds, we retain 8705 files, which are then individually z-transformed for normalization. From each of the 8705 files, we cut out 5 random time-intervals ('blocks') with a length of 0.2 seconds, corresponding to 8820 samples. To the resulting 43525 blocks we apply the same pre-processing as with the sleep data: each block is Fourier-transformed (FFT), the modulus of the complex Fourier components is calculated, and only the first 784 components with the lowest frequencies are retained. We then use an autoencoder (a) for unsupervised training and a perceptron (b) for supervised training on these 43535 data vectors of length 784. The parameters of the models are exactly as with the MNIST data set, which also had 10 classes. Above, we show MDS projections of the data distributions in different layers of the network, with data classes marked by colors. The degree of class separability is quantified by the GDV in the titles of the MDS plots. Additionally, we show example input patterns (as 28x28 pixel arrays) for each data class, together with their representations in the smallest network layer 3 (as 4x4 pixel arrays). In the case of the autoencoder, we also show the reconstructed patterns. We find a clear enhancement of the clustering (the GDV values fall from -0.032 in layer 0 to -0.136 in layer 3) in the four subsequent layers of the perceptron. For the autoencoder, we find a very small cluster enhancement in the first three layers (GDV falls from -0.032 in layer 0 to -0.035 in layer 2), which however disappears in layer 3. This behavior is very similar to the one observed with the sleep EEG data set.
